# 

**Published:** 1897-03

**Authors:** 


### MARRIAGE.

January 27, 1897, at Rock Island, Canada, Erastus P. Ball, D.V.S., graduate of McGill College, class of 1890, to Miss Florence Caswell, of Stanstead, Canada.

---

The *Jersey Bulletin* is printing weekly a series of letters on the value and dangers of feeding cotton-seed products. A writer, in the issue of February 10th, condemns it severely as a contributor to barrenness in cows and as a frequent cause of abortion.

### IMPORTANT VETERINARY ASSOCIATION MEETINGS.

Pennsylvania State Veterinary Medical Association, Philadelphia, March 2d and 3d; California State Veterinary Medical Association, San Francisco, March 19th; and New Hampshire Veterinary Medical Association.

### PERSONAL.

Veterinarian G. Howard Davison is one of the directors of the Dutchess County Agricultural Society of New York State.

"External Diseases of the Hog, and their Treatment," was the subject of an address by William Belshaw, Seneca, at the January meeting of the Kansas Board of Agriculture. Veterinarian C. J. Sihler, of Kansas City, addressed the above meeting on the "Necessity for Meat-inspection."

Dr. N. S. Mayo addressed the Kansas Swine-breeders' Association, at Topeka, in January, on "State Control of Contagious Diseases."

Prof. A. Smith, of Toronto, will be one of the judges at the coming Boston horse-show.

The veterinary inspectors selected for the Boston horse-show in April are Drs. F. H. Osgood, Thomas Blackwood, W. L. Labaw, Austin Peters, and L. H. Howard.

Dr. A. W. Clement has been appointed a member of the committee of racing of the Pimlico Driving Club, of Baltimore, Md.

Veterinarian T. L. Swift, of Attleboro, Mass., holds the official position of milk-inspector for Attleboro, North Attleboro, and Falmouth.

Dr. J. W. Ferguson, of Bay City, Mich., will deliver a course of lectures to the Horseshoers' Association of that city.

Dr. A. Drinkwater, of Rochester, N. Y., is lecturing to the shoers of that city on "Anatomy."

Western Vice-President Stalker, of the U. S. V. M. A., has been seriously ill at his home at Richland, Ia.

Veterinarian W. R. Cooper, of Quasqueton, Ia., graduate of the Veterinary Department of the Ames Agricultural College, Iowa, has received an appointment as assistant meat-inspector, and assigned to duty at Kansas City.

Dr. Alex. Glass, of Philadelphia, was an interested visitor to the national dog-show at New York, in February.

Dr. H. J. McClellan, of Bryn Mawr, Pa., has recently been a visitor to Baltimore, Md., as a prospective location for future professional labors.

Drs. Jas. W. Sallade, of Pottsville, and Otto Noack, of Reading, addressed the Berks County Agricultural Society on the subject of "Tuberculosis" in February.

Veterinarian Gilbert, of Pottstown, Pa., who had his leg broken in August last, and was otherwise injured, has almost fully recovered and resumed his professional work.

Dr. Chas. H. Higgins, who recently went to Jamaica, has returned to his home in Dover, Mass., not finding the climate of that country congenial.

Veterinarians Ridge, Minster, and Wand, of Bucks County, Pa., have been speakers on the subject of "Tuberculosis" at the several Farmers' Institutes held in that territory during December and January.

Dr. Lloyd, of Towson, Md., conducts a veterinary hospital in connection with his practice.

Dr. Ernest M. Hutchinson, graduate of the Chicago Veterinary College, recently of Jackson, Tennessee, is now located at San Francisco.

Dr. C. D. Smead, of Logan, N. Y., addressed the Empire State Sheep-breeders' Association, at Rochester, in January, on the "Diseases to which New York Sheep are Subject."

Dr. Tait Butler, of Agricultural College, Mississippi, was the purchaser of two Poland China boar hogs at the Springfield, Ill., sale in January.

Veterinarian Harrington has been elected Secretary of the South Boston Driving Association, recently organized.

Dr. C. C. McLean, of Meadville, Pa., has reconstructed his hospital, enlarging it to a considerable extent.

Dr. M. E. Knowles reports the animals distressingly healthy in Montana.

Dr. D. C. Stanton, of Factoryville, Pa., has been on the sick-list for some time past.

Dr. Jas. T. McAnulty is making a thorough canvass of the Keystone State in the interest of the horseshoers' bill, which is presented on another page of this issue of the JOURNAL.

Dr. John R. Mohler, of Philadelphia, graduate of the Veterinary Department of the University of Pennsylvania, has received an appointment under the Bureau of Animal Industry, and has been assigned a position along the border established as the Texas-fever line.

Dr. U. G. Houck, of Berwick, Pa., graduate of the Veterinary Department of the University of Pennsylvania, has received an appointment under the Bureau of Animal Industry, and has been assigned to duty at Chicago.

Prof. Sweetapple, of Toronto, Canada, has been a victim of the "grippe," but we are glad to report his convalescence.

Dr. Harry Walters, of Wilkesbarre, has been confined to the house by illness and unable to pursue his professional duties.

Veterinarian Schwarzkopf, of Chicago, Ill., has sufficiently recovered from the injuries received by a trolley-car injury to be at his post of duty.

Dr. W. H. Dalrymple, of Louisiana, has been elected secretary of the State Agricultural Society and secretary-treasurer of the State Stock-breeders's Association, a new organization in the Creole State.

Dr. H. Clay Glover officiated as veterinarian to the Westminster Kennel Club Exhibition at Madison Square Garden in February.

Veterinarian O. C. Farley has recently fitted up in an elaborate manner an office on Broadway, New York City.

Dr. James B. Rayner, of West Chester, Pa., has been seriously ill from a series of carbuncles.

Veterinarian J. A. Pearson, of Chicago, Ill., was a visitor to the JOURNAL office in February.

The many friends of Dr. S. E. Weber will be glad to learn that he is in greatly improved health, and looking forward to a return to his books and investigations.

Veterinarian S. C. Tremainie, of Bridgeton, N. J., takes a keen interest in the homing-pigeon, and was recently elected vice-president of the Homing Club of that city.

Veterinarian Oglesby, of Bonham, Tex., has been seriously ill, but we are glad to learn of the early prospects of his return to his professional studies.

Veterinarian J. E. Ryder was a strong second in the bidding for the famous hackney stallion "Matchless," at the sale in New York. It is said he represented the Duke of Marlborough.

Dr. P. I. Kershner, of Fleetwood, Pa., has received an appointment as inspector under the Bureau of Animal Industry, and has been assigned to duty along the Texas-fever line.

